# Involvement of IL17A, IL17F and IL23R Polymorphisms in Colorectal Cancer Therapy

**DOI:** 10.1371/journal.pone.0128911

**Published:** 2015-06-17

**Authors:** Inés Omrane, Imen Medimegh, Olfa Baroudi, Hager Ayari, Walid Bedhiafi, Nejla Stambouli, Marwa Ferchichi, Nadia Kourda, Yves-Jean Bignon, Nancy Uhrhammer, Amel Mezlini, Karim Bougatef, Amel Benammar-Elgaaied

**Affiliations:** 1 Laboratory of Genetics, Immunology and Human Pathology, Faculty of Sciences of Tunis, University Tunis El Manar, Tunis, Tunisia; 2 Laboratory of Anatomy and Cytopathology of the Charles Nicolle Hospital, Tunis, Tunisia; 3 Laboratory of Diagnostic and Molecular Genetics, Centre Jean Perrin, Clermont Ferrand, France; 4 Medical Oncology Department of the Institute Salah Azaiez, Tunis, Tunisia; Johns Hopkins Hospital, UNITED STATES

## Abstract

IL23/IL17 pathway plays an important role in the development of inflammatory bowel diseases (IBD). In general, the genes encoding the cytokines are genetically polymorphic and polymorphisms in genes IL23R and IL17 have been proved to be associated with its susceptibility to inflammatory diseases as well as cancer including colorectal cancer. Moreover, it has been shown that these interleukins are involved in anti-tumor or pro-tumor effects of various cancers. Previously, we showed that there is a significant association between IL17A, IL17F and IL23R polymorphisms as well as the occurrence of colorectal cancer and the clinical features of the disease. The purpose of the present work is to investigate an association between IL17A, IL17F and IL23R polymorphisms in 102 Tunisian patients with colorectal cancer treatment. The association was analyzed by statistical tools. We found that patients with mutated genotypes of IL17A G197A SNP could be a risk factor for the inefficiency of chemotherapy and radiotherapy. Unlike IL17F variant, patients with wild type genotypes require surgery and adjuvant chemotherapy. On the one hand, we found no evidence that supports a significant association between IL23R polymorphism and the combined genotypes of these three genes and the colorectal cancer treatment. On the other hand, we showed that there is an important interaction between IL17A/IL17F polymorphisms and the stage of the disease as well as its treatment. Finally, patients with IL17F wild type genotype highlighted that there is a valid longer OS without all treatments and with radiotherapy and a neoadjuvant chemotherapy. In contrast, we observed that there are no relationships between IL17A, IL23R and the survival of these patients neither with nor without the treatment. Our results suggest that polymorphisms in IL17A and IL17F genes may be a predictive source of colorectal cancer therapy type. Therefore, IL17F may serve as an independent prognostic factor for overall survival in patients with colorectal cancer.

## Introduction

Cytokines are parts of the extracellular signaling network that controls every function of the innate and specific immune responses by operating in anautocrine or paracrine manner. They are similar to hormones but they may be distinguished in particular by a more pleiotropic action and production involving a greater number of cell types. Taking into account their important role in immune responses, their therapeutic value in several contexts of immunological and infectious diseases seems obvious.

Th17 cells were described initially in 2005 as CD4 + cells secreting IL-17 [1]. Several studies have shown that pro-tumor and/ or anti-tumor functions of IL17 and IL23[2, 3] are cytokines that necessarily maintain the Th17 phenotype through its receptor IL23R[4]. IL17 is involved in the pathogenesis of many chronic inflammatory diseases[5–7]. This cytokine is considered as being an important mediator in an inflammation-associated cancer[8–11]. The IL17 induces the recruitment of immune cells in peripheral tissues. This response requires the activation of NF-kB after the commitment of IL17 to its receptor IL17R. IL17 also leads to the induction of many pro-inflammatory factors, including TNF-α, IL6 and IL1 beta[12]. There are two forms of IL 17: the IL17A and IL17F that act through a complex of two chains of IL17RA and IL17RC receptor.

IL17A had significantly increased peripheral blood and tissues levels from a variety of cancer patients. In contrast, IL17F was down-regulated in human colonic cancer tissues. Furthermore, genetic studies have revealed the presence of polymorphisms in the genes IL17A / F and IL23R which are associated with inflammatory bowel disease and some cancers such as bladder, breast, uterus and gastric cancer [6, 13–20]. Through case / control studies, we have recently showed that unlike IL17F rs763780 and IL23R rs10889677 polymorphisms, IL17A G197A polymorphism is associated with its susceptibility to colorectal cancer. Indeed, the mutated allele A of IL17A G197A polymorphism increases the risk of colorectal cancer[21, 22]. This result shows that most patients have the mutated allele (A 31,5%) in contrast to healthy subjects (GG 17,4%) suggesting that IL17A/AA genotype could be considered as a susceptibility factor for developing colorectal cancer (p = 0,002; OR 2,45 (1,43–4,11)) [23]. In addition, our results showed that polymorphisms of these three genes are associated with clinical data and the disease severity. Hereafter, we suggest that IL17A G197A, IL17F rs763780and IL23R rs10889677 polymorphisms are associated with the development and progression of colorectal cancer[21, 22].

It has already been reported that IL-17 producing cells can facilitate the development of colorectal carcinoma by promoting angiogenesis, the production of VEGF, and by tumor cells. Moreover, the modulation response of IL-17F may inhibit tumor angiogenesis and enhance the inflammatory response of the host to tumorigenesis [24]. For this purpose, the IL17A/F has been suggested as a new prognostic indicator in patients with colorectal cancer and could be considered as a new therapeutic target for colorectal cancer.

In our previous studies, we showed that the IL17A G197A, IL17Frs763780 and IL23Rrs10889677 polymorphisms are associated with the tumor location, especially with colon cancer [21]. Indeed, we found that IL17A as a wild type genotype could protect against colon cancer unlike mutated genotype that could increase the susceptibility of colon cancer[22]. Here, we analyzed the association between these polymorphisms, colon cancer and rectum cancer separately in order to confirm our results. Moreover, this study aims at evaluating the possible interaction between IL17A G197A, IL17F rs763780 and IL23Rrs10889677 polymorphisms as well as the treatment of colorectal cancer in Tunisian population such as the overall survival of patients with and without treatment.

## Material and Methods

### Subjects

A group of patients/control is collected from the Salah Azaiez hospital and Charles Nicolle hospital of Tunis (the cosmopolite population of Tunis- Tunisia). Patients include102 unrelated sporadic CRC cases (45 women, 55 men, with age range 58 ± 14) with no family cancer histories. They were classified on the bases of their histopathological profiles. These Patients received oral and written information about the study and gave their written consent. This study was approved by the Clinic Research Ethics Committee of “Institut Pasteur” of Tunis (Tunisia).

### DNA extraction and polymorphisms genotyping

The study of the association between polymorphisms in *IL 17A*, *IL17F* and *IL23R* genes with their susceptibility to colorectal cancer as well as clinical data of patients has been reviewed previously. In brief, the genomic DNA was extracted from peripheral blood leukocytes using conventional proteinase K digestion and the phenol/chloroform extraction method. A NanoDrop (ND-1000) is used to quantify DNA. IL17A/F and IL23R variants in patients and controls subjects were genotyped using specific primers for each polymorphism. PCR products were then analyzed using a fluorescent—based restriction fragment length polymorphism method as reported previously[21, 22].

### Statistical analysis

The data were analyzed using SPSS software (version 11.5.). Significance of the association was determined by Pearson’s chi-squared test χ^2^, Fisher’s exact test and Anova test. Time duration related to events was calculated as the difference between primary diagnosis and either the date of the clinical assessment where the respective event occurred or the last clinical assessment in case of censoring. While survival probabilities were graphically assessed by the Kaplan Meier method (including a log-rank test for inference in the figures). A value of p < 0,05 was considered significant.

## Results

Genotypes and alleles frequencies of IL17A, G197A IL17F rs763780 and IL23R rs10889677 variants were shown in Table 1.

**Table 1:** Characteristics of study subjects.

GenotypesIL17AG197A	Control	Cases	GenotypesIL17F	Control	Cases	GenotypesIL23R	Control	Cases
	n = 139(%)	n = 102(%)		n = 137(%)	n = 100(%)		n = 137(%)	n = 100(%)
**GG**	95(68,34)	48(47,05)	**AA**	98(71,5)	72(72)	**CC**	56(40,9)	12(12)
**GA**	38(34,53)	51(40,19)	**AG**	38(27,73)	27(27)	**AC**	63(46)	48(48)
**AA**	6(4,31)	3(2,95)	**GG**	1 (0,72)	1(1)	**AA**	18(13,1)	40(40)
**Gallelefrequency**	0,826	0,685	**Aallelefrequency**	0,85	0,85	**Callelefrequency**	0,53	0,55
**Aallelefrequency**	0,174	0,315	**Gallelefrequency**	0,15	0,15	**Aallelefrequency**	0,46	0,45
P = 0,002OR2,45(1,43–4,11)[22]			P = 0,96[21]			P = 0,92[21]		

To confirm that IL17F rs763780and IL23R rs10889677 polymorphisms were associated with the tumor location, we evaluate the relationship between colon cancer susceptibility and these two polymorphisms. On the one hand, We showed that IL17F AG+GG genotypes were more frequent in controls than in patients with colon cancer (p = 0,03OR 0,45 (0,21–0,98)). On the other hand, we found that IL23R AC+AA genotypes were more frequent in patients with colon cancer than in controls (p = 0,02 OR 2,11 (1,09–4,09)). However, there is no significant difference between the genotypes of rectal cancer patients and controls (Table 2). These results suggest that IL17F rs763780 and IL23R rs10889677 polymorphisms were rather associated with colon cancer than with rectal cancer. Indeed, IL17F AG+GG mutated genotypes could protect against colon cancer unlike IL23R AC+AA mutated genotypes that could increase the susceptibility of colon cancer (Table 2).

**Table 2:** Association between IL17F/IL23R polymorphisms and colon cancer.

Genotypes		COLONGROUP		P-Value
		Casesn = 65(%)	Controln = 137(%)	
**IL17Frs763780**	**AA**	55(84,61)	98(71, 53)	**0,03**
	**AG/GG**	10(15,38)	39(28,46)	**OR0,45**(0,21–0,98)
**IL23Rrs10889677**	**CC**	16(24,61)	56(40,87)	**0,02**
	**AC/AA**	49(75,38)	81(59,12)	**OR2,11**(1,09–4,09)

We investigate the association between IL17A G197A, IL17F rs763780 andIL23R rs10889677 polymorphisms and treatments of CRC patients which were stratified according to a surgery, neoadjuvant chemotherapy, adjuvant chemotherapy and a preoperative radiotherapy. We found that the majority of patients with wild type genotype AA of IL17F rs763780 were submitted to a radical surgery and an adjuvant chemotherapy (Table 3). We observed a significant association between IL17F polymorphism, surgery (p = 0,013; RR 0,7 (0,5–0,97)) and adjuvant chemotherapy treatment (p = 0,006; RR 0,69 (0,52–0,93)). Therefore, patients having IL17F wild type genotype GG require chemotherapy. In fact, the risk of a bad outcome is decreased, meaning that surgery and chemotherapy were likely to be beneficial effect in patients with IL17F polymorphism. On the basis of the relative risk and the relative risk reduction (RRR), chemotherapy could allow a relative risk reduction of CRC by 31%. The relative risk reduction of CRC in patients group under radiotherapy was 30% (Table 3). However, we did not find any significant association of this variant with a preoperative radiotherapy and a neoadjuvant chemotherapy (Table 3). In addition, we found that CRC patients with mutated genotypes GA/AA of IL17AG197Awere submitted to chemotherapy and radiotherapy (Table 3). In fact we proved that there is a significant association of IL17A polymorphism with chemotherapy (p = 0,028 RR 1,57 (1,06–2,32)) and preoperative radiotherapy (p = 0,01 RR 1,90 (1,08–3,33)) (Table 3). Indeed, this polymorphism could be a risk factor for chemotherapy and radiotherapy resulting in the inefficiency of the treatment.

**Table 3:** Association between IL17A, IL17F and IL23R polymorphisms and CRC treatments.

IL17AG197A				IL17FA7488G			IL23R		
Genotypes	GG	AG/AA	p value	AA	AG/GG	p value	CC/AC	AA	p value
**Surgery**									
**palliative and non-palliative surgery**	13	12	0,3	18	14	**0,013**	31	1	0,054
**radical surgery**	35	42		56	14	**RR** * **0,7**(0,50–0,97)**RRR** * **30%**	57	11	
**Neoadjuvant chemotherapy**									
**No**	39	36	0,07	55	20	0,47	63	11	0,12
**Yes**	9	18		19	8		25	1	
**Adjuvant chemotherapy**									
**No**	19	11	**0,028**	23	17	**0,006**	38	2	0,07
**Yes**	29	43	**RR 1,57**(1,06–2,32)	51	11	**RR0,69**(0,52–0,93)**RRR 31%**	50	10	
**Preoperative radiotherapy (dose in Gy)**									
**No**	38	30	**0,01**	55	20	0,47	64	10	0,34
**Yes**	10	24	**RR1,90**(1,08–3,33)	19	8		24	2	

*Relative risk (RR) = Event rate (treatment)/Event rate (Placebo).

**Relative risk reduction (RRR) = 1- relative risk x 100.

Finally, we have found that there is no link between IL23Rrs10889677 polymorphism and the different types of colorectal cancer treatment (Table 3). In order to see the additive effect of these three polymorphisms on CRC treatment, we analyzed the association between the combined genotypes of IL17A, IL17F and IL23R and each data. There is no evidence that supports the presence of a significant association between the combined genotypes of these three genes and colorectal cancer treatment (Table 4).

**Table 4:** Association between combined genotype of IL23R/IL17A and IL17F polymorphisms and CRC treatments.

Combined Genotypes				
IL23R/IL17A/IL17F	WT/WT	WT/MUT	MUT/MUT	P-Value
**Surgery**				
**palliative and non-palliative surgery n = 32**	9	23	0	0,77
**radical surgery n = 65**	19	45	1	
**Neoadjuvant chemotherapy**				
**No n = 72**	22	49	1	0,62
**Yes n = 25**	6	19	0	
**Adjuvant chemotherapy**				
**No n = 40**	13	26	1	0,37
**Yes n = 57**	15	42	0	
**Preoperative radiotherapy (dose in Gy)**				
**No n = 73**	19	53	1	0,49
**Yes n = 24**	9	15	0	

We also studied the interaction between the TNM stage of CRC and the type of the treatment. We showed that the majority of patients with IL17A GA/AA genotypes, who were treated by chemotherapy and radiotherapy, are in advanced stage of the disease. In fact, we showed that there is a significant association between the late stage (TNM III/IV) of colorectal cancer, the chemotherapy (p = 0,001 RR 7 (1,81–27,07)) and radiotherapy (p = 0,0003 RR 8,36 (2,11–33,00)) in patients with AG/GG genotypes of IL17A gene (Table 5). In addition, we observed that the risk relative RR has increased approximately 5 times for a chemotherapy and about 4,5 times for a radiotherapy. Indeed, our result proved that there is an additive effect between IL17A AG/GG genotypes and the late stage of the disease on the risk of treatment inefficiency. We have also found a positive link between IL17F polymorphism, a chemotherapy (p = 0,011 RR 0,7 (0,62–0,89)) and a surgery (P = 0,023 RR 0,76 (0,62–0,93)) in CRC patients with a late stage (Table 5).

**Table 5:** Interaction between IL17A and IL17F polymorphisms, CRC treatments and Stage of the disease.

IL17A G197A		Stage	AA	AG/GG	p-value
**Chemotherapy**	No	I/II	15	7	0,59
		III/IV	24	29	
	Yes	I/II	7	2	**0,001**
		III/IV	2	16	RR7,0(1,81–27,07)
**Radiotherapy**	No	I/II	14	6	0,10
		III/IV	24	24	
	Yes	I/II	8	3	**0,0003**
		III/IV	2	21	RR8,36(2,11–33,00)
**IL17F A7488G**		Stage	GG	GA/AA	p-value
**Chemotherapy**	No	I/II	7	3	0,29
		III/IV	16	14	
	Yes	I/II	20	0	**0,011**
		III/IV	30	10	RR 0,7(0,62–0,89)
**Radiotherapy**	No	I/II	5	2	0,31
		III/IV	13	12	
	Yes	I/II	22	1	**0,023**
		III/IV	33	12	RR0,76(0,62–0,93)

**Fig 1:**
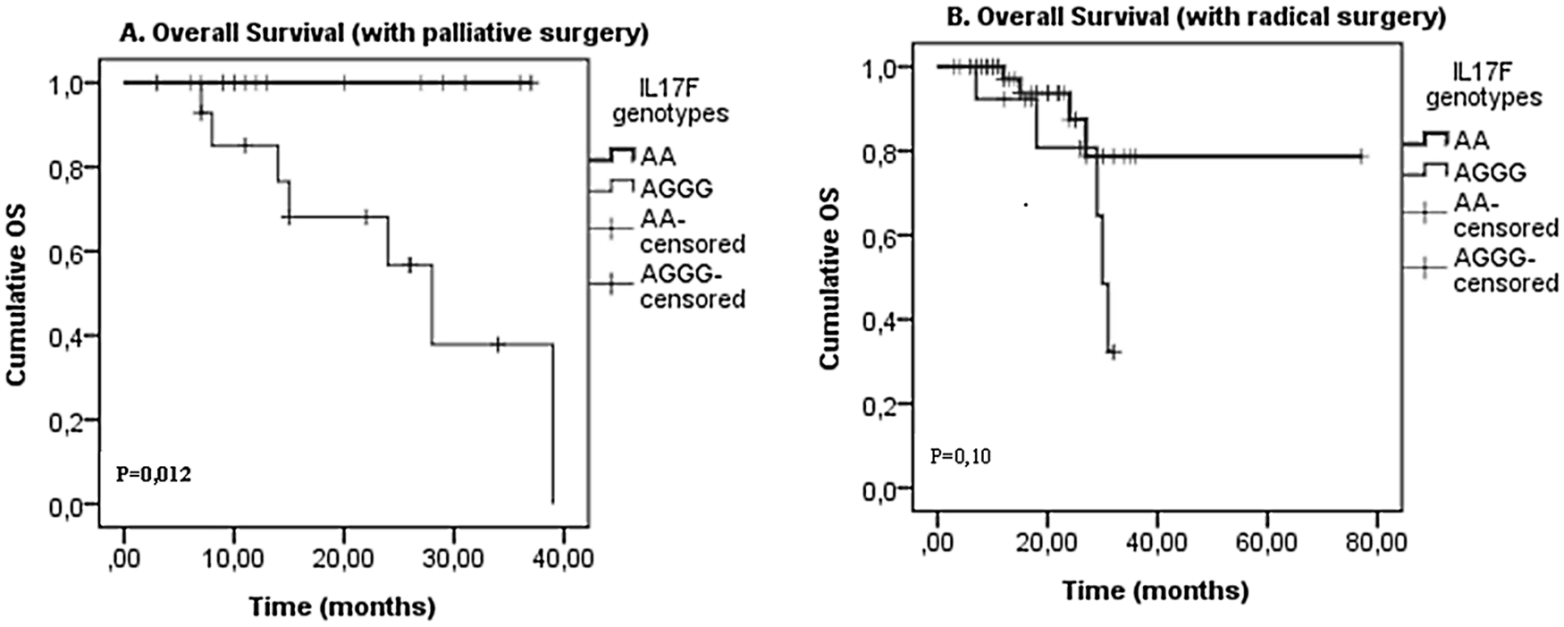
IL17F genotypes impact on overall survival stratified by surgery. (A) palliative surgery and (B) radical surgery; in CRC patients according to IL17F genotype (IL17F AA vs. IL17F AGGG). P-values from the log-rank test are indicated. OS: overall survival.

**Fig 2:**
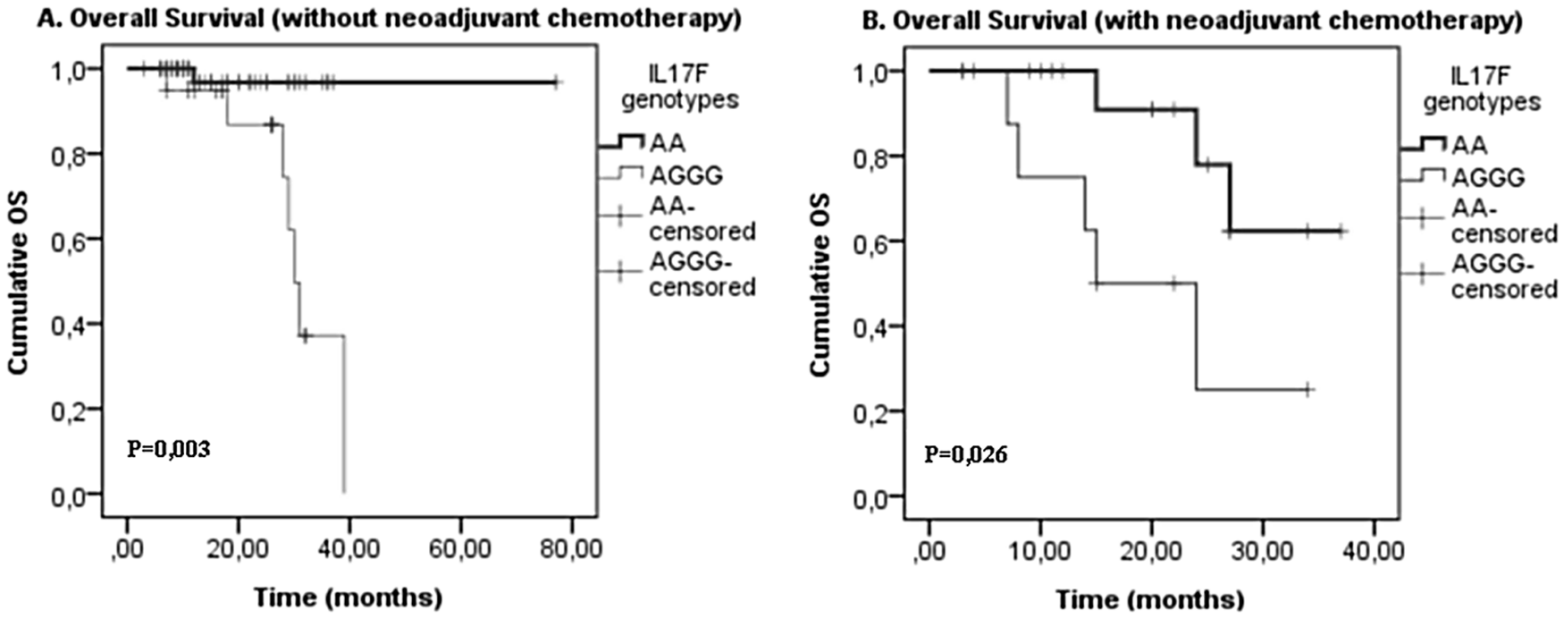
IL17F genotypes impact on overall survival stratified by neoadjuvant chemotherapy. (A) withoutneoadjuvant chemotherapy (**p = 0,003**) and (B) with neoadjuvant chemotherapy (**p = 0,026**); in CRC patients according to IL17F genotype (IL17F AA vs. IL17F AGGG). P-values from the log-rank test are indicated. OS: overall survival.

**Fig 3:**
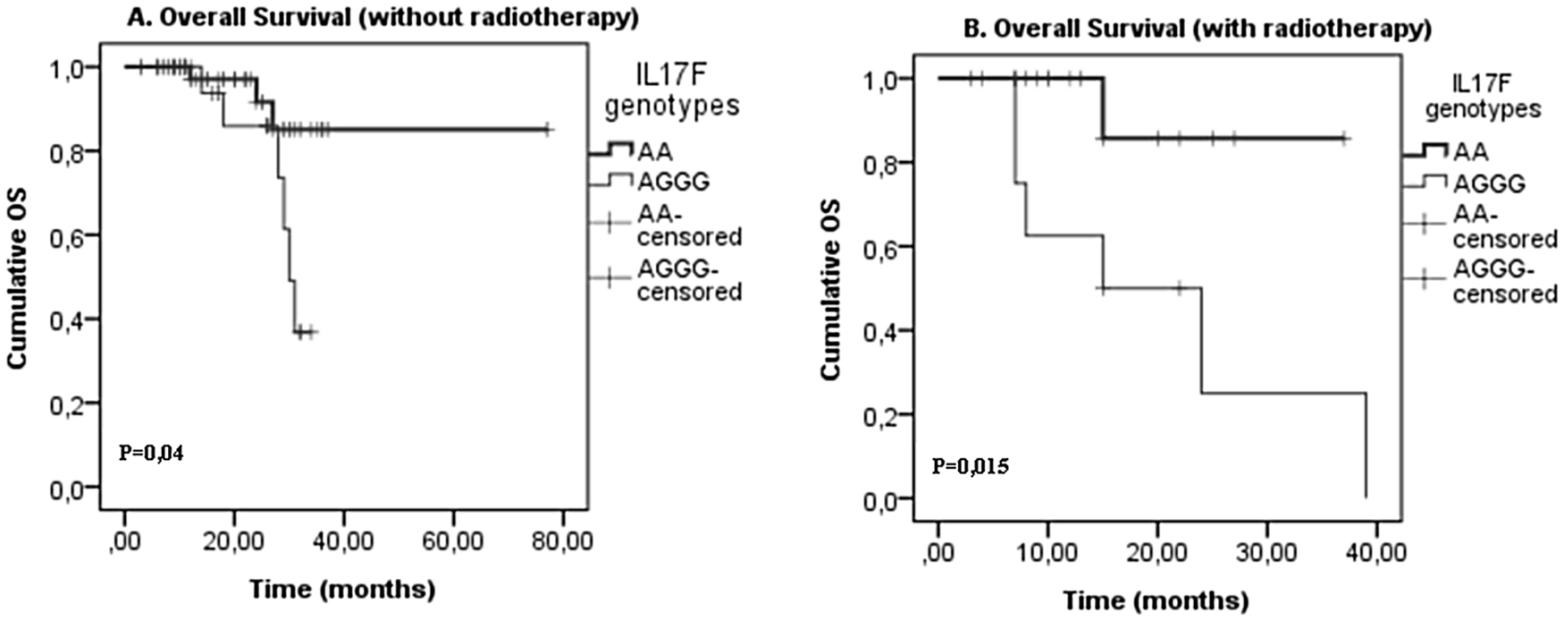
IL17F genotypes impact on overall survival stratified by radiotherapy. (A) without radiotherapy and (B) with radiotherapy; in CRC patients according to IL17F genotype (IL17F AA vs. IL17F AGGG). P-values from the log-rank test are indicated. OS: overall survival.

**Fig 4:**
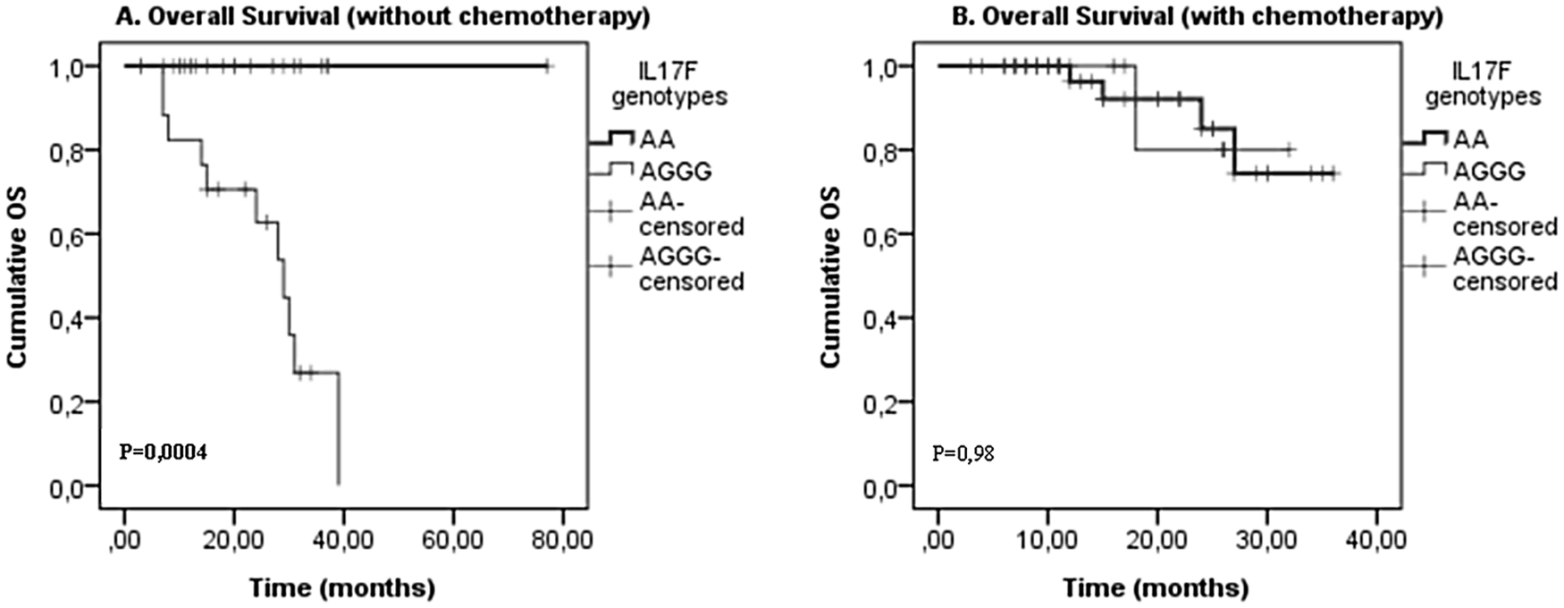
IL17F genotypes impact on overall survival stratified by chemotherapy. (A) Without chemotherapy and (B) with chemotherapy; in CRC patients according to IL17F genotype (IL17F AA vs. IL17F AGGG). P-values from the log-rank test are indicated. OS: overall survival.

Finally, our analysis revealed that patients with IL17F wild type genotype AA presented significantly longer OS, without a CRC treatment (chemotherapy (p = 0,0001),a surgery(p = 0,012), a radiotherapy (p = 0,04) and a neoadjuvant chemotherapy(p = 0,003)) (Figs 1, 2, 3 and 4). On the one hand, perceived that there is a significant association between IL17F AA genotype and longer OS in patients with a neoadjuvant chemotherapy (p = 0,026)(Fig 2)and a radiotherapy(p = 0,015) (Fig 3) in contrast to surgery and chemotherapy (Figs 1 and 4). On the other hand, we detected the absence of any significant association between IL17A and IL23R polymorphisms and survival of patients with or without treatment.

## Discussion

Recently, we hypothesized that Th17 cells can contribute to carcinogenesis and that IL17A, IL17F and IL23R polymorphisms can affect the susceptibility to colorectal cancer. In fact, we showed that IL17A G197A polymorphism was positively correlated with an increased risk of developing colorectal cancer. In addition, we showed that IL17F and IL23R polymorphisms were positively associated with colon tissue mostly associated to colon location of the tumor[21, 22]. Here we showed that IL23R variant could increase the susceptibility to colorectal cancer unlike IL17F polymorphism which could confer protection against this cancer. Other polymorphisms have also been shown to decrease cancer risk. Indeed, Slattery et al reported that the GG genotype of the 174 G/C IL6 polymorphism was associated with a significantly reduced risk of colon, but not rectal cancers[25]. High expression of IL-6 has been correlated with a poor survival in CRC patients and CC genotype of IL6 SNP was also significantly associated with a shorter survival time when compared with the heterozygous genotype CG[26–28]. For TNFα polymorphism, two alleles (a5 and a13) were associated with decreased risks of CRC[29, 30].

Our results can be confirmed by some other previous studies. Indeed, Z Tong et al found that VEGF (vascular endothelial growth factor) expression levels are increased in colon tissue in mice with colon cancer and IL17F deficient (IL17F - /-)[24]. Moreover, they showed that IL 17F was down-regulated in human colonic cancer tissues and that it could be a protective role in the development of colon cancer, possibly via an inhibition of tumor angiogenesis[24]. However, IL17A has been reported to stimulate angiogenesis in tumor[31]. Further, Kawaguchi et al revealed that the expression and/or activity of IL17F may be suppressed in IL17F rs763780 polymorphism and this variant is able to block IL8 induced by wild-type IL17F. Besides, they suggested that the IL17F rs763780 variant is a natural antagonist for the wild-type IL17F and may be a potential therapeutic target[32].

In light of these results, we additionally studied the association between polymorphisms in *IL17F*, *IL17A* and *IL23R* genes and different types of colorectal cancer treatment. We found interesting that IL17A G197A variant is associated with a poor prognosis for treatment according to an adjuvant chemotherapy (p = 0,028 RR 1,57 (1,06–2,32)) and a preoperative radiotherapy (p = 0,01 RR 1,90 (1,08–3,33)) (Table 3). Furthermore, we found that IL17F A7488G polymorphism is significantly associated with a good prognosis for an adjuvant chemotherapy (p = 0,006 RR 0,69 (0,52–0,93)) and a surgery (p = 0,013 RR 0,7 (0,5–0,97)) (Table 3). Indeed, the chemotherapy and surgery could reduce the relative risk of colorectal cancer frequency by 31% and 30% respectively in patients with IL17F variant. They suggest that this polymorphism could be predictive of the need for a surgery and chemotherapy. Other studies have shown that 3020insC and G908R polymorphisms of NOD2/CARD15 gene could be predictive of the need for first and subsequent surgeries in patients with Crohn's disease[33–36]. However, further studies have found that despite the significant association of these two variants with Crohn's disease progression or with CRC treatment, they may not be predictive markers due to their low frequencies in Spanish and Tunisia populations[23, 37]. IL23R polymorphism is not associated with any type of CRC treatment (Table 3). Conventional chemotherapy agents have a direct cytotoxic effect and may cause the death of cancer cells. However, the effectiveness of chemotherapeutic agents, such as anthracyclines and oxaliplatin is higher in immunocompetent murine models, compared to immunodeficient models[38, 39]. Furthermore, it was shown that the response to chemotherapy agents was reduced during lymphopenia[40, 41], and immunosuppression because it was predictive of a lower response to anthracyclines and oxaliplatin[40, 42]. These data support an immune response induced by chemotherapy agents against the tumor. Few studies have examined the role of IL17A / F in the treatment of cancer. Wedebye Schmidt *et al* suggested that blockade of both IL17A and IL17F attenuates the development of colitis in a T-cell transfer model of experimental colitis[43]. Leom P McLean et al showed that the treatment with antibodies against both IL17A and IL17F significantly enhanced colitis scores when compared with mice treated with a control antibody. They suggested that there is the presence of a potential therapeutic role for combined blockage of IL17A and IL17F in the treatment of inflammatory bowel disease, rather than blocking either cytokine alone[44]. Sarah Reppert et al suggested that local anti IL17A antibody therapy could be successful for the treatment of lung tumor and it is also efficient with the absence of Th1 specific factor T-bet in a murine model of lung adenocarcinoma[45]. Other studies have shown that Il17Apromotes proliferation and resistance to chemotherapeutic agents such as docetaxel by the ERK1/2 pathway in human breast cancer cells lines [46]. Furthermore, we showed that there is a significant interaction between IL17A and IL17F polymorphisms, treatments (surgery, chemotherapy and radiotherapy) and TNM stage of the disease in CRC patients (Table 5). In a breast cancer, it has been shown that there is the presence of a significant association between CYP2D6 polymorphism and early stage of the disease in women treated with tamoxifen[47]. Dan Su et al, showed that ERCC1 and iASPP polymorphisms were associated with a chemotherapy in patients with advanced non-small cell lung cancer [48]. A further study has shown that mutations in genes 17p, 18q and 20q and TP53 could add information to the Dukes sub-classification B and C in patients with colorectal cancer; it may have an impact on the choice of the treatment [49]. However, no study has focused on the effect of immunity genes polymorphisms on treatment in combination with clinical data.

Finally, we showed that patients with IL17F wild type genotype AA have significantly longer OS without all types of the treatment and have longer DFS with a radiotherapy and a neoadjuvant chemotherapy (Figs 2 and 3). In fact, Xi Chen et al showed that IL17 expression is an independent prognostic factor in both overall and disease-free survival in non-small cell lung cancer[50]. In addition, Jing-Ping Zhang et al noted that intratumoral IL17producing cell density was associated with an overall survival and a disease-free survival. Therefore, this increased expression is correlated with a poor survival in hepatocellular carcinoma patients[51]. Other studies have also demonstrated that IL17 could be an independent prognostic factor for an overall survival in colorectal cancer [52]. Moreover, it has been also shown that 5’-TSER and 3’-TSUTRpolymorphisms of thymidylate synthase (TS) gene have an efficient impact on the survival of colorectal cancer patients receiving adjuvant 5-fluorouracil. Indeed, TS polymorphism may serve as an independent prognostic marker in selecting CRC patients with a poor prognosis, and it may be useful to examine if these patients would benefit from an alternative therapy[53].

To sum up, IL17F appears to play protective roles in colon cancer unlike IL17A polymorphism that increases risk of CRC. In addition, CRC patients with IL17F wild type genotypes need a surgery and chemotherapy. However, patients with IL17A mutated genotype may decrease the effectiveness of the treatment. A significant relationship was found between IL17A/IL17F polymorphisms, the treatment and the late stage of the disease. Therefore, we proved the presence of a positive link between IL17F variants and OS of patients with and/or without any treatment. We suggest thatIL17A and IL17F genes may be predictive of colorectal cancer treatment and IL17F may be an independent prognostic factor for an overall survival in colorectal cancer. Therefore, the regulation of the expression levels of IL17F and IL17A could be evaluated. Further investigations are now needed to clarify the involvement of IL17A and IL17F polymorphisms in colorectal carcinogenesis and its treatment in order to answer the famous question whether IL17A and IL17 F are reliable therapeutic targets.
